# Protective effect of *Rhus coriaria* fruit extracts against hydrogen peroxide-induced oxidative stress in muscle progenitors and zebrafish embryos

**DOI:** 10.7717/peerj.4144

**Published:** 2017-12-12

**Authors:** Fadia Najjar, Francine Rizk, Gilles Carnac, Rim Nassar, Sara Jabak, Anatoly Petrovich Sobolev, Yara Bou Saada, Marwan El Sabban, Aline Hamade

**Affiliations:** 1 Departments of Biology, Chemistry and Biochemistry, Laboratoire d’Innovation Thérapeutique, Faculty of Sciences, Lebanese University, Fanar, Lebanon; 2 PhyMedExp, University of Montpellier, INSERM U1046, CNRS UMR 9214, Montpellier, France; 3 Istituto di Metodologie Chimiche, CNR, Laboratorio di Risonanza Magnetica Nucleare “Annalaura Segre”, Monterotondo, Rome, Italy; 4 UMR 8256, CNRS, Université Pierre et Marie Curie, Paris, France; 5 Department of Anatomy, Cell Biology and Physiological Sciences, American University of Beirut, Beirut, Lebanon

**Keywords:** Sumac *Rhus coriaria* extract, Myoblast (LHCN-M2), Aging, NMR, Antioxidants, Zebrafish embryos

## Abstract

**Background and Purpose:**

Oxidative stress is involved in normal and pathological functioning of skeletal muscle. Protection of myoblasts from oxidative stress may improve muscle contraction and delay aging. Here we studied the effect of *R. coriaria* sumac fruit extract on human myoblasts and zebrafish embryos in conditions of hydrogen peroxide-induced oxidative stress.

**Study Design and Methods:**

Crude ethanolic 70% extract (CE) and its fractions was obtained from sumac fruits. The composition of sumac ethyl acetate EtOAc fraction was studied by ^1^H NMR. The viability of human myoblasts treated with CE and the EtOAc fraction was determined by trypan blue exclusion test. Oxidative stress, cell cycle and adhesion were analyzed by flow cytometry and microscopy. Gene expression was analyzed by qPCR.

**Results:**

The EtOAc fraction (IC_50_ 2.57 µg/mL) had the highest antioxidant activity and exhibited the best protective effect against hydrogen peroxide-induced oxidative stress. It also restored cell adhesion. This effect was mediated by superoxide dismutase 2 and catalase. Pre-treatment of zebrafish embryos with low concentrations of the EtOAc fraction protected them from hydrogen peroxide-induced death *in vivo*. ^1^H NMR analysis revealed the presence of gallic acid in this fraction.

**Conclusion:**

*Rhus coriaria* extracts inhibited or slowed down the progress of skeletal muscle atrophy by decreasing oxidative stress via superoxide dismutase 2 and catalase-dependent mechanisms.

## Introduction

Skeletal muscles constitute about 40% of total body mass in humans and are essential for many functions, such as locomotion, postural maintenance, metabolic homeostasis, respiration, and thermoregulation (Frontera & Ochala, 2015). The healthy state of muscles is critical for maintaining physical activity and overall energy balance; therefore muscle damage should be rapidly repaired. Precursor cells play a major role in muscle repair and renewal (Collins et al., 2005; Peault et al., 2007; Sacco et al., 2008; Zammit et al., 2004).

Skeletal muscle can be also affected by diseases. Muscular dystrophies (MDs) are a group of inherited disorders characterized by progressive muscle wasting and weakness. Related clinical manifestations vary in symptoms and severity, ranging from muscular fatigability, muscular weakness, and muscular pain, leading to restrictive respiratory insufficiency, motor disabilities and orthopedic problems (Emery, 2002; Flanigan, 2012). MDs are multifactorial pathologies in which nutritional, endocrine, metabolic and immunological components contribute to muscle depletion. In this context, skeletal muscle wasting is associated with increased oxidative stress (Moylan & Reid, 2007; Powers & Jackson, 2008).

Oxidative stress is induced by the imbalance between the generation and removal of reactive oxygen species (ROS). Radicals derived from oxygen represent the most important class of ROS (Halliwell & Gutteridge, 2015; Sies, 2007; Sies & Cadenas, 1985). ROS have long been associated with both physiology and pathology of skeletal muscle (Clanton, Zuo & Klawitter, 1999; Reid et al., 1992); ROS level is crucial for the regulation of muscle contraction and is associated with muscle fatigue. Skeletal muscle is affected by the age-related loss of function, whether directly or because of aging of other organs that supports its functionality (Bross, Storer & Bhasin, 1999; Shefer et al., 2006). The “Free Radical Theory of Ageing” was described for the first time in 1956 (Harman, 1956) and the accumulation of oxidative damage to lipids, proteins and DNA occurring with age induces skeletal muscle aging (Hekimi, Lapointe & Wen, 2011). Many studies suggest that enhancing the organism antioxidant capacities may delay the aging process (Gems & Doonan, 2009; Willett, 2006). However, other studies showed that antioxidant supplements so far tested seem to offer no improvement over a well-balanced diet, possibly because of the choice of the substances tested or of an excessive dosage (Dolara, Bigagli & Collins, 2012).

*Rhus coriaria L.* (Tanner’s Sumac or Sicilian Sumac) is a wild plant growing mainly in the Mediterranean countries, Iran and Afghanistan (Özcan & Haciseferogullari, 2004). Extracts from the fruit of *Rhus coriaria* contain high levels of phenolic compounds, organic acids and terpenoids (Abu-Reidah et al., 2015). Furthermore, earlier studies showed that the fruits are rich in oleic and linoleic acids, vitamins as well as minerals (Gabr, El-Metwally & Al-Ghadir, 2014; Kosar et al., 2007; Kossah et al., 2009; Powers & Jackson, 2008).

Sumac extracts possess a potential antiviral, antimicrobial, antifungal, antioxidant and hypolipidemic activities (Ali-Shtayeh, Al-Assali & Jamous, 2013; Aliakbarlu, Mohammadi & Khalili, 2014; Kossah et al., 2013; Onkar, Mohammed & Nida, 2011). Moreover, sumac is beneficial in the treatment of inflammation, diarrhea, oral diseases, dysentery and strokes (Rayne & Mazza, 2007) and the antioxidant activity of fruit and leaves extracts from sumac has been demonstrated (Aliakbarlu, Mohammadi & Khalili, 2014; Candan & Sökmen, 2004). Chakraborty et al. (2009) showed that sumac was able to protect human DNA, rat tissues and organs from oxidative stress-induced damage (Chakraborty et al., 2009). Antioxidant activities of sumac extracts may improve cell viability in several progressive diseases reinforcing the defenses against free radical species. The biological activity of sumac plants is attributed to their content of several antioxidant agents, phenolics compound being a major fraction (Gabr, El-Metwally & Al-Ghadir, 2014; Kosar et al., 2007).

Zebrafish (*Danio rerio*) is a convenient animal model in investigating embryo-toxic and teratogenic compounds or food materials of potential value. The embryonic development of zebrafish is similar to that of the higher vertebrates, including humans (Howe et al., 2013; MacRae & Peterson, 2003; Teraoka, Dong & Hiraga, 2003; Zon, 1999). Thus, chemicals potentially toxic for zebrafish embryos could have similar effects on other vertebrate embryos. The features of this research model include similarities with mammals in physiological pathways, in functional domains of many genes associated with diseases, high rate of fecundity, external fertilization (allowing embryos to be exposed to drugs), rapid development, optical transparency of embryos and availability of genetic tools for research purposes (Alestrom, Holter & Nourizadeh-Lillabadi, 2006; Ekker & Akimenkko, 1991; Hong, 2009; Kimmel et al., 1995; Parng et al., 2002). A particular advantage for screening natural products such as herbal medicines, using this model, is the relatively small quantities of extracts used during the test.

Here we have assayed the activity of Sumac fruit extracts using free radical scavenging activity (DPPH) and the β carotene-bleaching tests. The effects of these extracts in conditions of H_2_O_2_-induced oxidative were studied on cultured human myoblasts and zebrafish embryos.

## Materials and Methods

### Collection and preparation of sumac extracts

Fresh fruit from *Rhus coriaria L.* plant was collected from South Lebanon, dried at room temperature until weight stabilization then pulverized.

The powdered sample (50 g) was mixed with 70% ethanol and left at room temperature in the dark for 7 days with non-continuous stirring. The extract was filtered through paper filter and the ethanol was evaporated under reduced pressure. Residual water was lyophilized to obtain a crude extract (CE) stored at 4 °C. The lyophilized powder was then taken up in 75 mL of water and undergone liquid-liquid extraction successively with several solvents: n-hexane, dichloromethane (CH_2_Cl_2_) and ethyl acetate (EtOAc). The different fractions hex (oil), CH_2_Cl_2_ (oil), EtOAc (powder) and aqueous (aq, powder) were obtained after evaporation of the organic solvents and lyophilization of water.

### Determination of antioxidant activity

#### DPPH free radicals scavenging activity

The DPPH radical scavenging activity of the different extracts was measured according to the method of Yen & Chen (1995) with slight modifications (Brand-Williams, Cuvelier & Berset, 1995) and as described by Auezova et al. (2013). Serial dilutions of the sumac extracts were prepared in ethanol. The basic procedure was to add an aliquot (1 mL) of the test sample to 1 mL of DPPH solution prepared with ethanol (0.15 mM). The mixture was vortexed for 1 min and then left to stand at room temperature for 30 min in the dark. The absorbance was measured at 517 nm using UV-Visible VWR spectrophotometer. The scavenging activity (SA) was calculated as follows: SA (%) = [1 − (A_sample_ −  A _sample blank_)/A_control_] × 100. The sample solution (1 mL) plus ethanol (1 mL) was used as a sample blank and DPPH solution (1 mL) plus ethanol (1 mL) was used as a negative control. Catechin and ascorbic acid were used as positive controls. Stock solutions of catechin and ascorbic acid (0.8 mg/mL) were diluted with ethanol to give concentrations ranging from 1.5 to 20 µg/mL. All measurements were performed in duplicate.

#### *β*-carotene bleaching test

The antioxidant activities of samples assayed by the linoleic acid-β-carotene system were measured according to the method described by Koleva et al. (2002) with slight modifications and as described by Auezova et al. (2013). Beta-carotene (10 mg) was dissolved in 10 mL chloroform, and 0.2 mL of this solution was mixed with linoleic acid (20 mg) and Tween-40 (200 mg). After removal of chloroform by evaporation under vacuum at 40 °C, 50 mL of distilled water were added slowly to the semi-solid residue under vigorous stirring to form an emulsion, which was always prepared just before each experiment. A 96-well plate was loaded with 50 µL per well of the samples or positive controls (catechin) and 200 µL of the emulsion. One final concentration was tested (50 µg/mL), and ethanol was used as a blank. The absorbance values were read at 450 nm on a multi-well spectrophotometer (ELx800 Bio-Tek). The starting time of the reaction (*t* = 0 min) is considered when the emulsion is added. Then, the plate was covered with a film and stored at 30 °C for 3 h; the absorbance was measured every 30 min. The antioxidant activity of the extracts was evaluated as the percentage of inhibition of the bleaching of β-carotene using the following formula: [(1 − (Δextract_*t*0−*t*_/Δcontrol _*t*0−*t*_)] * 100. All samples were assayed in duplicates.

### NMR studies

The NMR experiment was done as described elsewhere by Sobolev et al. (2014). Briefly, samples for NMR were prepared by dissolve in 5–10 mg of an extract in a deuterated solvent (methanol-d_4_ or the mixture of acetone-d_6_/D_2_O). The NMR spectra of extracts were recorded at 27 °C on a Bruker AVANCE 600 NMR spectrometer operating at the proton frequency of 600.13 MHz and equipped with a Bruker multinuclear *z*-gradient inverse probe head. ^1^H spectra were acquired by adding 128 transients with a recycle delay of 3 s. The experiments were carried out by using a 90°pulse of 10 µs, 32K data points.

### Human myoblast cell culture

LHCN-M2 is a line of human skeletal myoblasts derived from satellite cells from the pectoralis major muscle of a 41-year-old Caucasian male heart transplant donor (Zhu et al., 2007). Cells were grown as undifferentiated myoblasts in DMEM AQ media (Lonza, Basel, Switzerland) supplemented with 15% Fetal Bovine Serum (FBS, Sigma-Aldrich, St Louis, MO, USA) and 1% penicillin-streptomycin (Lonza, Basel, Switzerland) at 37 °C in a humidified atmosphere with 5% CO_2_ and 95% air. The cells were usually split in a 1:3 ratio (33.33%, passage 1) when they reached about 60% confluence. The culture was passaged by removing the media, washing with phosphate buffered saline (PBS, Sigma, St Louis, MO, USA) and separating the cells from their support with trypsin-EDTA (Gibco, New York, NY, USA). Cells were then centrifuged at 900 rpm (150 g) for 5 min and the pellet was suspended in 3 mL of fresh media in order to be seeded in a flask (Corning, New York, NY, USA) for passaging or well culture plates for the appropriate experiments. Doubling time for myoblast cell cultures is set to 10 to 15 h in order to avoid senescence.

### *In vitro* assay for cytotoxic activity

The cytotoxicity of sumac was determined by the trypan blue exclusion test. Cells were seeded in a 24-well-plate with a concentration of 20 × 10^3^ cells/ well. The cells were left to adhere for 24 h before their exposition to different concentrations of the plant extracts (1, 3, 10, 30, 60 and 90 µg/mL) for 24 h, 48 h and 72 h. At each time point, the media was removed; the cells were washed with PBS, split with trypsin-EDTA and centrifuged at 900 rpm for 5 min. The pellet was suspended in 100 µl fresh media. The cell suspension was diluted (1:1, v/v) with trypan blue to reach 0.4%. Each condition was done in duplicates and three independent experiments were performed.

The effect of sumac extracts on H_2_O_2_-induced oxidative stress was determined by trypan blue exclusion test as described above. Cells were treated with 1 and 3 µg/mL of CE and EtOAc fraction; 48 h after the treatments, 75 µM of H_2_O_2_ (Sigma-Aldrich, St Louis, MO, USA) were added to the cells for 24 h.

### ROS detection with dihydroethidium staining

ROS production was monitored by fluorescent microscopy using dihydroethidium (DHE) staining. This assessment was obtained by measuring the ROS production in cell culture samples treated with different concentrations of EtOAc and crude extracts. After 48 h oxidative stress was induced with 75 µM of H_2_O_2_ for 24 h. Cells were then washed with PBS, 300 µL of DHE (10 µM) was added to each well and then incubated for 15 min. After incubation, DHE was removed and replaced with 4% of formaldehyde for fixation. Finally, cells were observed with a confocal microscope (LSM).

### Cell cycle analysis by flow cytometry

To assess the effect of sumac extracts on LHCN-M2 cell cycle distribution after inducing the oxidative stress, 4 × 10^4^ cells were seeded in 12-well plates. After 24 h of incubation, the media was removed and replaced with a new one containing 1 µg ml^−1^ of CE extract, 1 and 3 µg ml^−1^ of EtOAc fraction. After 48 h, 75 µM of H_2_O_2_ was added; 24 h later, cells were treated with trypsin then centrifuged at 1,500 rpm for 5 min. The pellet was washed with ice-cold PBS, centrifuged at 1,500 rpm for 5 min, suspended with ice-cold PBS and fixed using absolute ethanol.

Fixed cells were treated for 1 h with 200 µg/mL of DNase-free RNase A. 500 µL of PBS was added to 1 mg/mL of PI (Molecular Probes©, Invitrogen, Paisley, UK). Cells were incubated for 10 to 15 min in the dark and later centrifuged in order to eliminate the non-stained cells. Cells were suspended in 200 µL PBS in a flow tube (BD Falcon, Franklin Lakes, NJ, USA). A total of 10,000 gated events were acquired by flow cytometry (FACSAria, Becton Dickinson, Franklin Lakes, NJ, USA) representing the population of cells in each phase of the cell cycle. Subsequent data analysis and gating to determine the percentage of each cell cycle phase were done using FlowJo software. The experiment was repeated three times.

### Cell adhesion assay

Myoblasts were either left untreated or pre-treated with 1 or 3 µg/mL of EtOAc fraction. After 48 h, cells were subsequently trypsinised, seeded in 24 well plates and oxidative stress was induced using 75 µM of H_2_O_2_ for 4 h. Non-adherent cells were removed by washing with PBS and the adherent cells were trypsinised, collected and counted.

### RNA extraction and quantitative real-time PCR (qRT-PCR)

LHCN-M2 cells were seeded in 6-well plates at a density of 8 × 10^4^. 24 h post-seeding, cells were either left untreated or pre-treated with 1 and 3 µg/mL of the EtOAc fraction. 48 h post-treatment, cells were treated with 75 µM of H_2_O_2_. At the appropriate time point, the media was removed, cells were washed with PBS and the plates were stored at −80 °C. Total RNA was extracted from the cells using Nucleospin RNA II kit (Macherey-Nagel, Düren, Germany) as per manufacturer’s instructions. RNA purity and concentration were measured using NanoDrop™ spectrophotometer and then RNA was stored at −20 °C for subsequent cDNA synthesis.

cDNA was prepared with 1 µg of total RNA using Revertaid 1st strand cDNA synthesis kit (Fermentas, Thermo Scientific, Pittsburgh, PA, USA). The expression of various genes was analyzed by quantitative reverse transcriptase polymerase chain reaction (RT-qPCR) using the IQ SYBR GreenSupermix (Bio-Rad Laboratories, Pleasanton, CA, USA) in a CFX96 system (Bio-Rad Laboratories). Primers were designed using LightCycler design 2.0 (Roche Diagnostics, Indianapolis, IN, USA) and were tested for homology with other sequences using BLAST from NCBI database. Real-time PCR products were amplified using specific primers for myoD, myf5, myogenin, Gpx3, catalase, SOD2, and GAPDH (Table 1). PCR parameters consist of a pre-cycle at 95 °C for 3 min followed by 40 cycles consisting of 95 °C for 10 s, 60°C for 30 s, and 72 °C for 30 s. A final extension at 72 °C for 5 min was then performed followed by a melting curve, starting at 55 °C with a gradually increased temperature by steps of 0.5 °C to arrive at 95 °C. The calculation method used was the standard curve method. The fluorescence threshold cycle value (Ct) was obtained for each gene and normalized to that obtained for the GAPDH housekeeping gene in the same sample to normalize for discrepancies in sample loading. All experiments were carried out in duplicates and repeated three times.

**Table 1:** Primers designed for real-time PCR experiment (retrieved from Primer-Blast^®^).

Gene of interest	Primer sequence
myoD	F: 5′-ACAACGGACGACTTCTATGAC-3′ R: 5′-TGCTCTTCGGGTTTCAGGA-3′
myf5	F: 5′-CATGCCCGAATGTAACAGTC-3′ R: 5′-CCCAGGTTGCTCTGAGG-3′
myogenin	F: 5′-ACCCCGCTTCTATGATGG-3′ R: 5′-ACACCGACTTCCTCTTACACA-3′
GPx3	F: 5′-CGGGGACAAGAGAAGTCG-3′ R: 5′-CCCAGAATGACCAGACCG-3′
SOD 2	F: 5′-GGAGATGTTACAGCCCAGATAG-3′ R: 5′-CAAAGGAACCAAAGTCACG-3′
Catalase	F: 5′-CTGACTACGGGAGCCAC R: 5′-TGATGAGCGGGTTACACG
GAPDH	F: 5′-TGGTGCTCAGTGTAGCCCAG-3′ R: 5′-GGACCTGACCTGCCGTCTAG-3′

### Origin and maintenance of parental zebrafish

Adult wild-type zebrafish (*Danio rerio*) (Tübingen background; 3–5 cm) of both sexes were obtained from a specialized commercial supplier UMS AMAGEN CNRS INRA (France) and were used after ethical approval. Animals were housed in groups of 15 fishes in 5L thermostated tanks at 28 ± 2 °C, kept under constant chemical, biological and mechanical water filtration and aeration. Fish were maintained under a 14–10 h day/night photoperiod cycle, fed three times a day with commercial flakes (TetraMin™, Blacksburg, VA, USA) and supplemented with live brine shrimp. Embryos were obtained from natural spawning that was induced in the morning by turning on the light. A collection of embryos was completed within 30 min. Embryos were maintained in a specific E3 medium (34.8 g NaCl, 1.6 g KCl, 5.8 g CaCl_2_ 2H_2_O, 9.78 g MgCl_2_ 6H_2_O).

### Waterborne exposure of zebrafish embryos to sumac extracts and H_2_O_2_

Tests on zebrafish eggs were performed according to OECD 203 (1992) and according to the OECD Guideline for Testing of Chemicals 210, Fish, Early Life Stage Toxicity Test (OECD, 1992). Fertilized eggs of zebrafish were sorted in the 12th stage (very late blastula), corresponding to 2.5–3-hour post fertilization (hpf). Only eggs of the same quality were used in the experiments. No spontaneous defects of development occurred in embryos after exceeding this stage of development and survival of embryos in the control conditions was 100%.

Zebrafish embryos were transferred to 24 well plates, 10 embryos per well and three replicates per concentration for each of the evaluated endpoints were used in all the exposures. Embryos were maintained in 2 mL E3 medium and were exposed to 1, 3, 10, 30, 60, 90 and 120 µg/mL of sumac CE and EtOAc fraction. All bioassays included a negative control and a 0.3% DMSO control. Fish embryonic development was observed directly using a binocular microscope Leica. The following teratogenicity criteria were observed: incidence and extent of morphological abnormalities, hatching time and the number of hatched fish.

### Effect of sumac extracts on H_2_O_2_-induced oxidative stress

4–6 hpf embryos were transferred to individual wells of a 24 well plate at a density of 10 embryos/well and maintained in 2 mL of embryos medium containing CE and EtOAc sumac extracts at different concentrations (1 and 3 µg/mL) for 24 h. Embryos were then treated with H_2_O_2_ for 2 h at a concentration of 2.10^−2^ mol/L. Vitamin C has been used as a positive control at a concentration of 100 µM. Embryos viability was measured constantly using a stereoscope at intervals of 1 h after treatment.

### Statistical analysis

Experiments studying the cell viability and cell cycle distribution were performed in triplicates. Results were expressed as mean values ± SD and the corresponding error bars are displayed in the graphical plots. Statistical analysis was performed using the ANOVA test followed by post hoc tests of Duncan or Turkey for more precision. The study of the evolution of 2-time points was performed using the paired samples Student *t*-test. Differences were considered significant for p values less than 0.05. All analyses were done using the GraphPad Prism software (version 7.0).

## Results

### Sumac crude extract and an ethyl acetate fraction show *in vitro* antioxidant activity

We used 70% ethanol to extract dried ground to powder seeds of sumac. The crude ethanolic extract was obtained with a yield of 42.40% relative to the dry plant. Through solvent–solvent partitioning with hexane, dichloromethane and EtOAc, four fractions were obtained from the crude extract. Among the fractions, the highest yield was observed in the aqueous fraction (64.16%), followed by the EtOAc fraction (33.53%), dichloromethane (1.24%) and hexane (1.07%) ones.

Antioxidant activity of sumac extracts was evaluated by DPPH free radical scavenging and β-carotene bleaching assays. The DPPH free radical method determined the antiradical power of antioxidants (Brand-Williams, Cuvelier & Berset, 1995). For IC_50_ values, sumac extracts depleted the initial DPPH concentration by 50% within 30 min.

The free radical scavenging activities of the extract and fractions tested in this study are shown in Table 2. The highest antioxidant activity was exerted by the EtOAc fraction (IC_50_ 2.57 ± 0,51 µg/mL). The other extracts had lower antioxidant activities: Crude (IC_50_ 6.44 ± 0,35 µg/mL) >  Hexane (IC_50_ 18.66 ± 0,28 µg/mL) > Aqueous (IC_50_ 39.4 ± 3,98 µg/mL) > CH_2_Cl_2_(IC_50_ 43.66 ± 4,91 µg/mL).

**Table 2:** Antioxidant activity of *Rhus coriara*.

Sample	DPPH IC50 (µg/mL)	BCB Inhibition (%)
Crude (CE)	6.44 ± 0,35	47.01
Hexane	18.66 ± 0,28	36.75
CH_2_Cl_2_	43.66 ± 4,91	30.94
Ethyl acetate (EtOAc)	2.57 ± 0,51	69.23
Aqueous	39.4 ± 3,98	31.62
Catechin	2.4 ± 0,10	64.10
Ascorbic acid	2.5 ± 0,10	Nd

Results obtained after β-carotene bleaching assay were consistent with the data obtained with the DPPH test. Thus, EtOAc extract showed the greatest antioxidant activity (69.23% of inhibition of β-carotene bleaching at 50 µg/mL) superior to the inhibition capacity of the catechin positive control (64.10%) at the same concentration. Crude extracts also exhibited a significant antioxidant power (47.01%). Aqueous, hexane and CH_2_CL_2_ extracts showed the weakest activity potential in this assay (Table 2).

### Sumac extract is enriched in gallic acid and gallotanins

**Figure 1:**
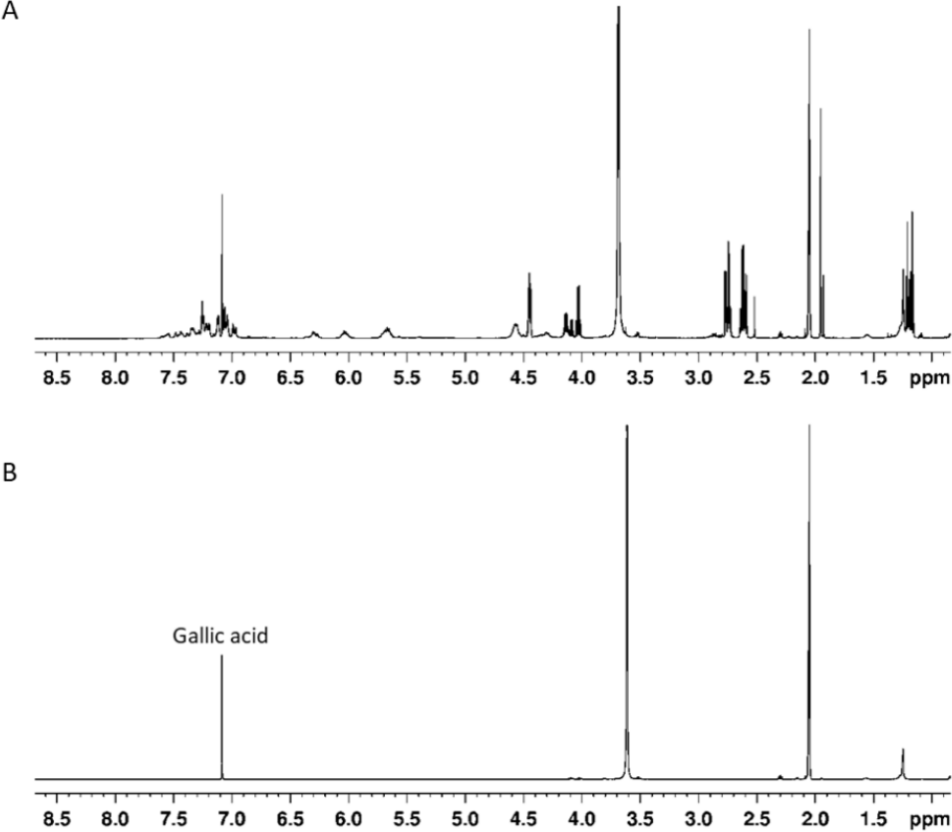
(A) ^1^H NMR spectrum of sumac ethylacetate extract in acetone-d_6_/D_2_O (9:1) v/v mixture, (B) ^1^H NMR spectrum of gallic acid in acetone-d_6_/D_2_O (9:1) v/v mixture.

The composition of sumac ethyl acetate extract was studied by ^1^H NMR. The most intense ^1^H NMR signals at 7.08 ppm (in deuterated methanol) in the aromatic compounds’ region 7.0–7.7 ppm belongs to gallic acid. The major part of the remaining signals from the phenolic fraction can be assigned to gallotannins. This assignment was confirmed by the comparison of our data with those from the literature where several components of gallotannin fraction were chemically synthesized and characterized by NMR (Sylla et al., 2015). To compare our and published NMR data, the extract was dissolved in acetone-d_6_/D_2_O (9:1) mixture. Apart from aromatic signals, gallotannins show characteristic signals of esterified β-glucose at 6.30 ppm (CH-1), 6.03 ppm (CH-3), 5.67 ppm (CH-2, and CH-4) and 4.57 ppm (CH-5 and CH_2_-6). Other signals in 4.5–1.0 ppm region belong to malic acid (Fig. 1).

### Sumac fractions are not cytotoxic at low concentrations on LHCN-M2 cells

*In vitro* cytotoxicity test is mainly performed to screen for potentially toxic compounds that affect basic cellular functions. We measured cytotoxicity of the EtOAc fraction and crude extracts of sumac on human myoblast cell line LHCN-M2 using trypan blue exclusion assay.

**Figure 2:**
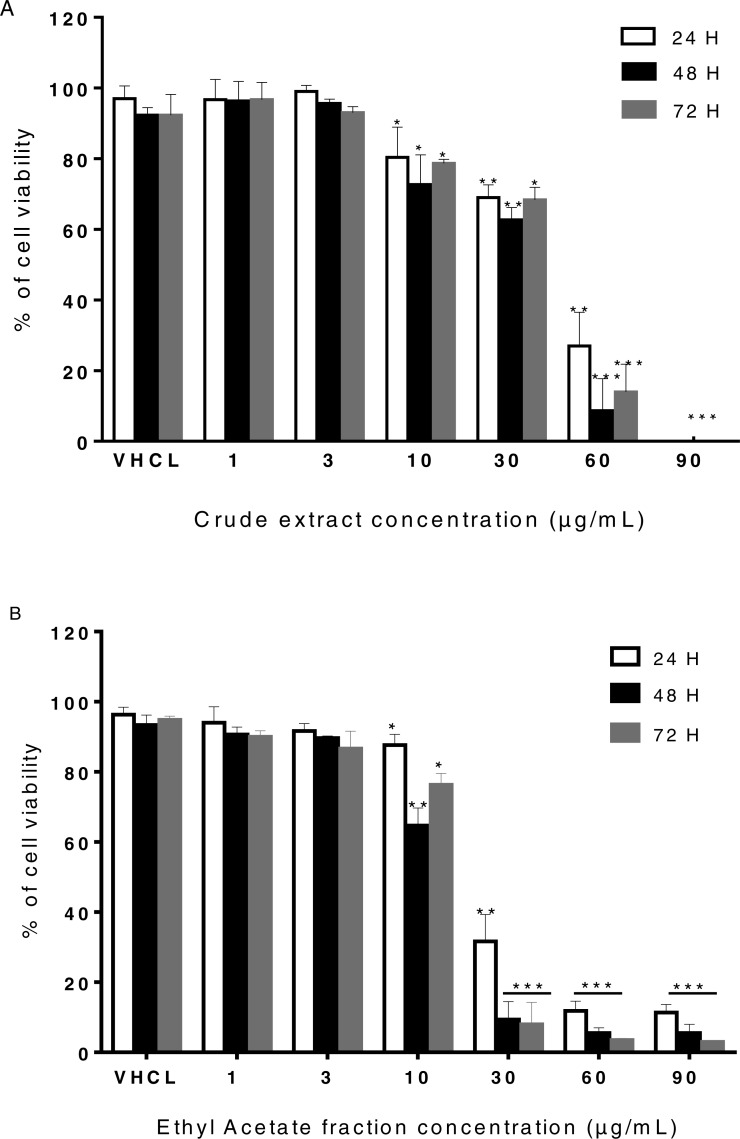
Cell viability of LHCN-M2 cells. (A) Cells were cultivated in the presence of CE (1, 3, 10, 30, 60 and 90 µg/mL) for 24, 48 and 72 h. (B) Cells were cultivated in the presence of EtOAc (1, 3, 10, 30, 60 and 90 µg/mL) for 24, 48 and 72 h. The results were expressed as a percentage of treated cells normalized to untreated control cells. Vehicle (VHCL) treated cells were used as the negative control. Mean values (% of control) with S.D. are indicated. This experiment was performed in duplicates and repeated three times. The significance (^∗^*p* ≤ 0.05, ^∗∗^*p* ≤ 0.01, ^∗∗∗^*p* < 0.001) of cell viability in treated cells with respect to the untreated cells (CTRL).

Neither of the two extracts was cytotoxic at low concentrations (<10 µg/mL), but the growth of LHCN-M2 cells decreased with increasing concentrations of each of these two extracts in a dose-dependent manner (Fig. 2).

As shown in Fig. 2A, at 60 and 90 µg/mL of crude extract, the maximal inhibition of cell viability reached 80 and 100% respectively after 24, 48 and 72 h of treatment (*p* ≤ 0.001). The treated-LHCN-M2 cells displayed a dose-dependent decrease in cell survival after 48 h and 72 h (for CE 1; 3; 10 and 30 µg/mL). Similarly, treatment of LHCN-M2 cells with EtOAc fraction significantly decreased cell viability reaching 80 to 100% when used at 30, 60 and 90µg/mL after 24, 48 and 72 h post-treatment (Fig. 2B).

Using data obtained from the trypan blue assay, IC_50_ was found to be higher than 30 µg/mL for the CE extract and higher than 10 µg/mL of the EtOAc fraction. Concentrations lower than 10 µg/mL for the CE and EtOAc fraction were used in subsequent assays.

### The crude extract and the ethyl acetate fraction protect human myoblast from H_2_O_2_ -induced oxidative stress at low concentration

**Figure 3:**
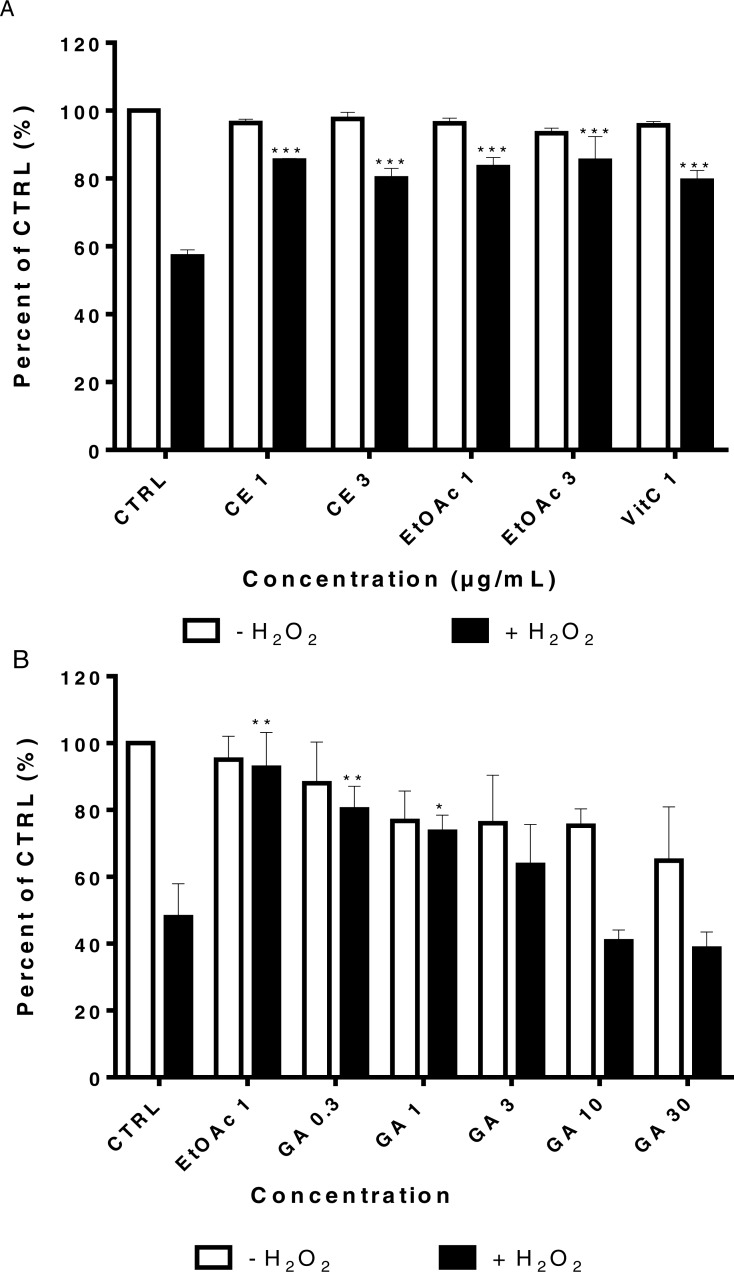
Viability of LHCN-M2 cells after pre-treatment with or without plant extracts for 48 h followed by exposure to hydrogen peroxide. Cells were cultivated in presence of (A) CE (1 and 3 μg/mL), EtOAc (1 and 3 μg/mL) and VitC (1 μ g/mL) or (B) gallic acid (1, 3, 10 and 30 μ M) for 48 h prior to stimulation with 75 µM H_2_O_2_ for 24 h. The results were expressed as a percentage of treated cells normalized to untreated control cells. Untreated cells were used as the negative control; VitC was used as positive control. Mean values (% of control) with S.D. are indicated. This experiment was performed in duplicate and repeated three times. The significance (^∗^*p* < 0.05, ^∗∗^*p* < 0.01, ^∗∗∗^*p* < 0.001) of cell viability in treated cells/+H_2_O_2_ with respect to the untreated cells/−H_2_O_2_.

LHCN-M2 cells were pre-incubated with or without CE extracts and EtOAc fraction (1 and 3 µg/mL) for 48 h then exposed to 75 µM H_2_O_2_ for 24 h at 37 °C. This concentration of H_2_O_2_ significantly decreased cell viability (*p* < 0, 001). Pre-treatment with the CE and the EtOAc fraction significantly protected LHCN-M2 cells from H_2_O_2_-induced oxidative stress when used at low concentrations (1 and 3 µg/mL), similarly to the positive control (1 and 3) µg/mL Vitamin C (VitC) (Fig. 3A). In parallel, the Fig. 3B showed the protective antioxidant effect of gallic acid at low concentration (0.3 µM), and this activity decreased due to the cytotoxic effect of this product at a higher concentration, superior to 10 µM.

**Figure 4:**
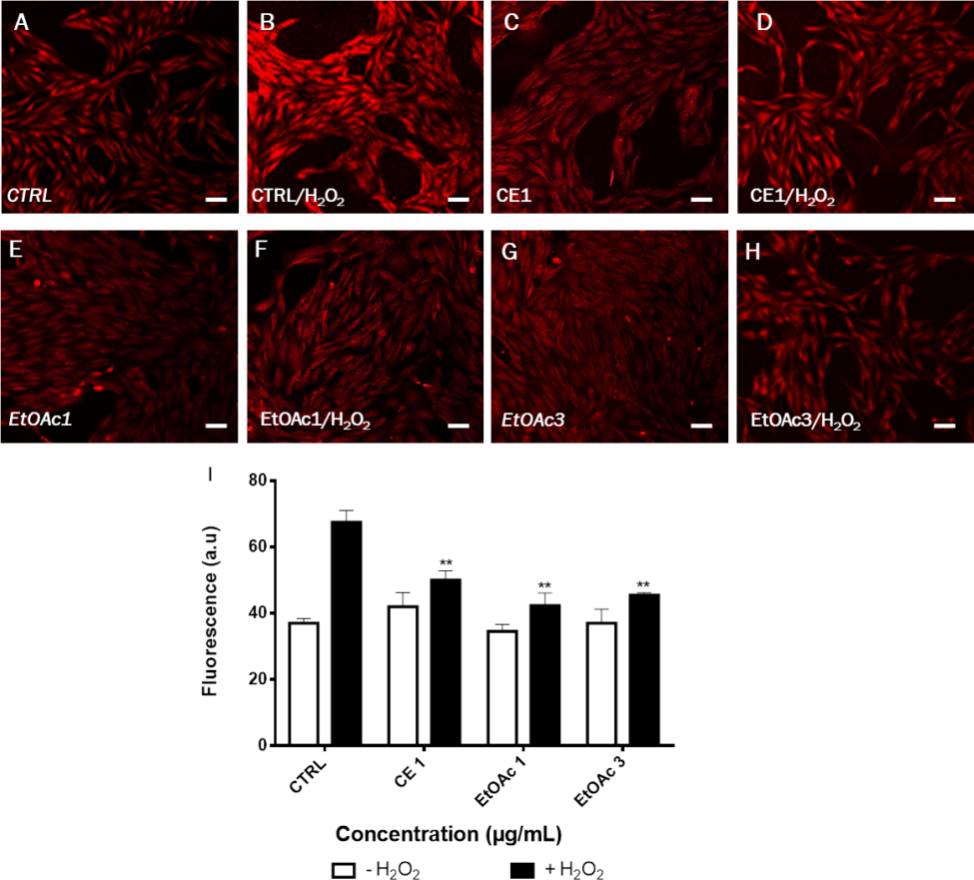
Assessment of ROS production in LHCN-M2 cells after pre-treated with sumac extracts and stimulated with 75 μM H_2_O_2_. (A–H), Representative DHE fluorescence staining of the oxidative stress. (I), Fluorescence was calculated and plotted on the graph for different concentrations of CE and EtOAc fractions of sumac using the ImageJ software. The significance (^∗∗^*p* ≤ 0.01) of fluorescence in treated cells/+H_2_O_2_ with respect to the untreated cells/−H_2_O_2_. Scale bar 20 μm.

In our study, DHE staining was used to assess the levels of ROS after treatment of LHCN-M2 cells with low concentrations of sumac extracts. As shown in Fig. 4, treatment with 75 µM H_2_O_2_ induced accumulation of ROS in untreated cells (high red fluorescence) whereas cells without H_2_O_2_ showed a low intensity of fluorescence (control). On the other hand, 1 µg/mL of CE extract, 1 and 3 µg/mL of EtOAc fraction significantly decreased the ROS level as compared to the H_2_O_2_-treated control (*p* < 0.05). The EtOAc fraction (1 and 3 µg/mL) exhibit the highest antioxidant effect on LHCN-M2 cells (Fig. 4I).

### Sumac extracts reduce cell cycle arrest in myoblasts subjected to oxidative stress

**Figure 5:**
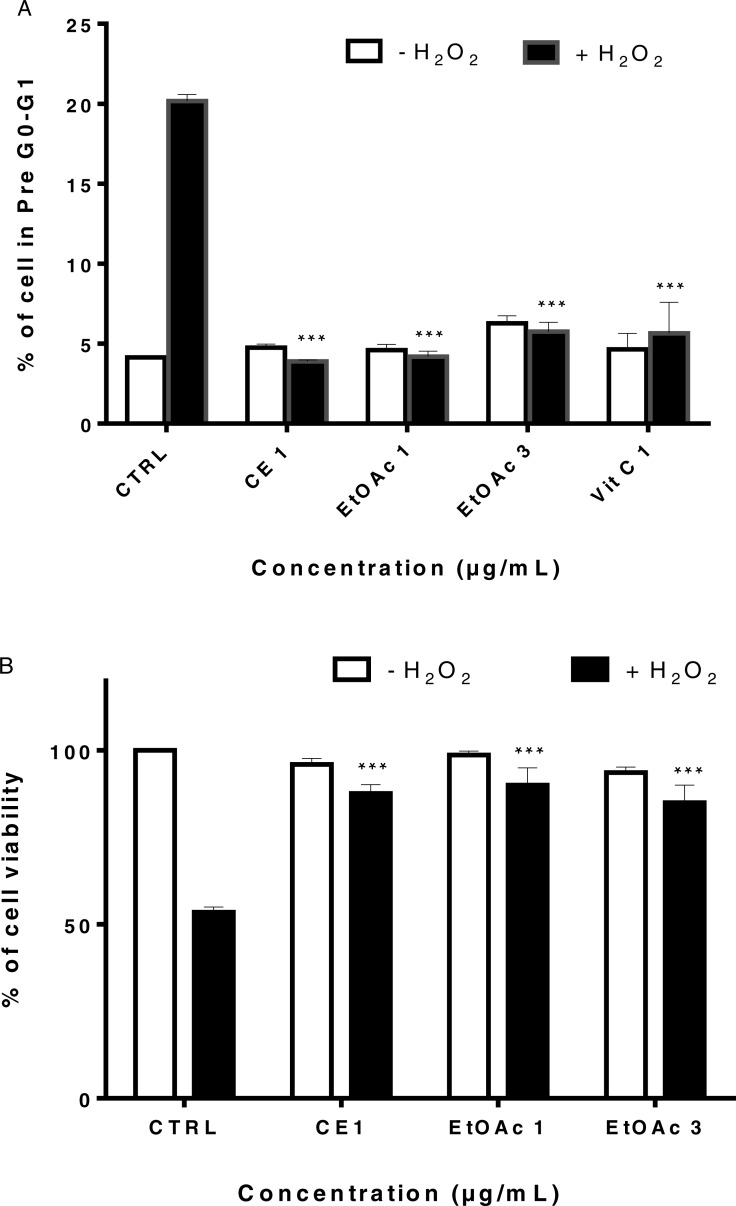
(A) Percentage of cells in pre G0/G1 of LHCN-M2 cells after pretreatment with sumac extracts and addition of H_2_O_2_. Cells were pre-treated with 1 and 3 μg/mL of the EtOAc fraction, 1 μg/mL of CE, 1 μ g/mL of VitC for 48 h, and incubated with 75 μM of H_2_O_2_. The histogram shows the percentage of cells of the total population at pre G0-G1. Values are means of three independent experiments. The significance (^∗∗∗^*p* ≤ 0.001) of cells in pre G0/G1 in treated cells/ +H_2_O_2_ with respect to untreated cells/−H_2_O_2_. (B) EtOAc fraction of sumac restores human myoblast adhesion under oxidative stress conditions. Cells were pre-treated with 1 and 3 μg/mL of the EtOAc fraction, 1 μg/mL of CE, 1 μg/mL of VitC for XX hours, and incubated with 75 μM of H_2_O_2_. The results of count were expressed as the percentage of treated cells normalized to untreated control cells. The untreated cells and the solvent treated cells were used as a negative control. Mean values (% of control) with S.D. are indicated. This experiment was performed in duplicates and repeated three times. The significance (^∗∗∗^*p* < 0.001) of cell viability in treated cells/ +H_2_O_2_ with respect to untreated cells/−H_2_O_2_.

Figure 5 illustrated the effect of sumac extracts on cell cycle arrest in LHCN-M2 cells treated with H_2_O_2_. Twenty percent of LHCN-M2cells treated with H_2_O_2_ underwent cell cycle arrest, whereas pretreatment of LHCN-M2 cells with 1 and 3 µg/mL of EtOAc fraction, 1 µg/mL of CE or 1 µg/mL of VitC reduced the percentage of arrested cells to 4 to 8% (Fig. 5A). Both CE and the EtOAc fraction significantly reduced cell cycle arrest in LHCN-M2 cells subjected to oxidative stress. The low concentrations of sumac CE and EtOAc fraction did not affect LHCN-M2 cell cycle (Supplemental Information 1).

### The crude extract and the ethyl acetate fraction restore myoblast adhesion impaired by H_2_*O*_2_

H_2_O_2_ is known to induce separation of cells from the substratum (Grossmann, 2002; Song et al., 2010); we examined the effects of H_2_O_2_ on myoblast adhesion in the presence of the sumac EtOAc fraction by using a quantitative adhesion assay. Cells were pre-treated for 2 days with CE extract at 1 µg/mL or EtOAc fraction at 1 and 3 µg/mL before being trypsinized, plated and immediately exposed to 75 µM of H_2_O_2_ for 4 h. Non-adherent cells were then removed and adherent cells were collected and counted by the trypan blue exclusion test.

As shown in Fig. 5B, H_2_O_2_ leads to a highly significant decrease in myoblast adhesion (*p* ≤ 0.001) and induces non-adherence in 50% cells while pre-treatment of LHCN-M2 cells with EtOAc fractions (1 µg/mL and 3 µg/mL) significantly restored myoblasts adhesion to around 90%. We have thus shown that pre-treatment of LHCN-M2 cells with CE extracts and EtOAc fraction prevented the deleterious effects of H_2_O_2_-induced oxidative stress in myoblasts and restored cells adhesion. Taking in consideration the low number of active molecules in the EtOAc fraction compared to the CE extract, the molecular study was conducted using only the low concentration of EtOAc fraction.

### SOD2 and catalase RNAs expression are activated by the ethyl acetate fraction

**Figure 6:**
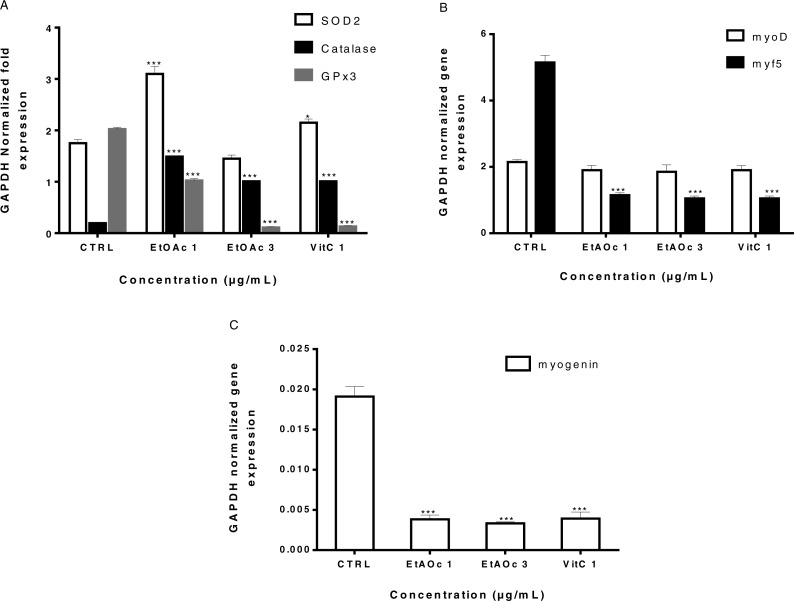
Expression of antioxidant genes GPx3, SOD, catalase and muscle determination and differentiation genes myoD, myf5 and myogenin in LHCN-M2 cells. The levels of Gpx3–SOD2 Catalase (A), myoD-myf5 (B) and myogenin (C). mRNA were analyzed using RT-qPCR 48 h after treatment with EtOAc fraction and VitC (A, B, C). Expression of GAPDH was used as an internal control to normalize the target gene levels. Experiments were performed twice. The significance (^∗^*p* ≤ 0.05, ^∗∗^*p* ≤ 0.01 & ^∗∗∗^*p* < 0.001) of gene expression in treated cells with respect to the untreated cells (CTRL).

We have previously shown that sumac extracts protected human myoblasts from H_2_O_2_-induced oxidative stress when pre-treated at least 48 h prior to H_2_O_2_ treatment. This may suggest an upregulation of genes encoding antioxidant enzymes. In order to identify genes that may mediate the anti-oxidant protective effect of sumac extracts, LHCN-M2 cells were treated for 2 days with either 1, 3 µg/mL of the EtOAc fraction or Vitamin C. mRNA levels of GPx3, SOD2 and catalase antioxidant genes were determined by RT-qPCR. We have observed increased levels of SOD2 (∼1.8 fold) in cells treated with 1 µg/mL of EtOAc fraction as compared to the untreated controls. Catalase level was also increased (∼7.6 fold) in all treated cells. The treatment induced a significant decrease in GPx3 expression (Fig. 6A). This result confirmed that SOD 2 and catalase might mediate the anti-cytotoxic effect of our extracts.

To investigate whether EtOAc fraction induces a change in the expression of the myoD gene family involved in muscle determination and differentiation, RT-qPCR was performed to evaluate the levels of myoD, myf5 and myogenin mRNAs in LHCN-M2 cells. In the case of myf5, a transcription factor engaged in muscle determination, a significant decreased was found in its expression in the case of both EtOAc fraction (1 and 3 µg/mL) and VitC (1µg/mL) (Fig. 6B). Expression of myogenin, a gene involved in muscle differentiation, was inhibited after EtOAc fraction (1 and 3 µg/mL) and VitC treatment (Fig. 6C).

Thus, SOD2 and catalase might mediate the antioxidant effect of EtOAc fraction at low concentrations. EtOAc fraction and VitC might inhibit or delay muscle differentiation. Our results are in agreement with the published data that treatment of myoblasts with antioxidants inhibited muscle differentiation (Zakharova et al., 2016).

### Low concentrations of sumac crude extract and the ethyl acetate fraction show no cytotoxic effect on zebrafish embryos

**Figure 7:**
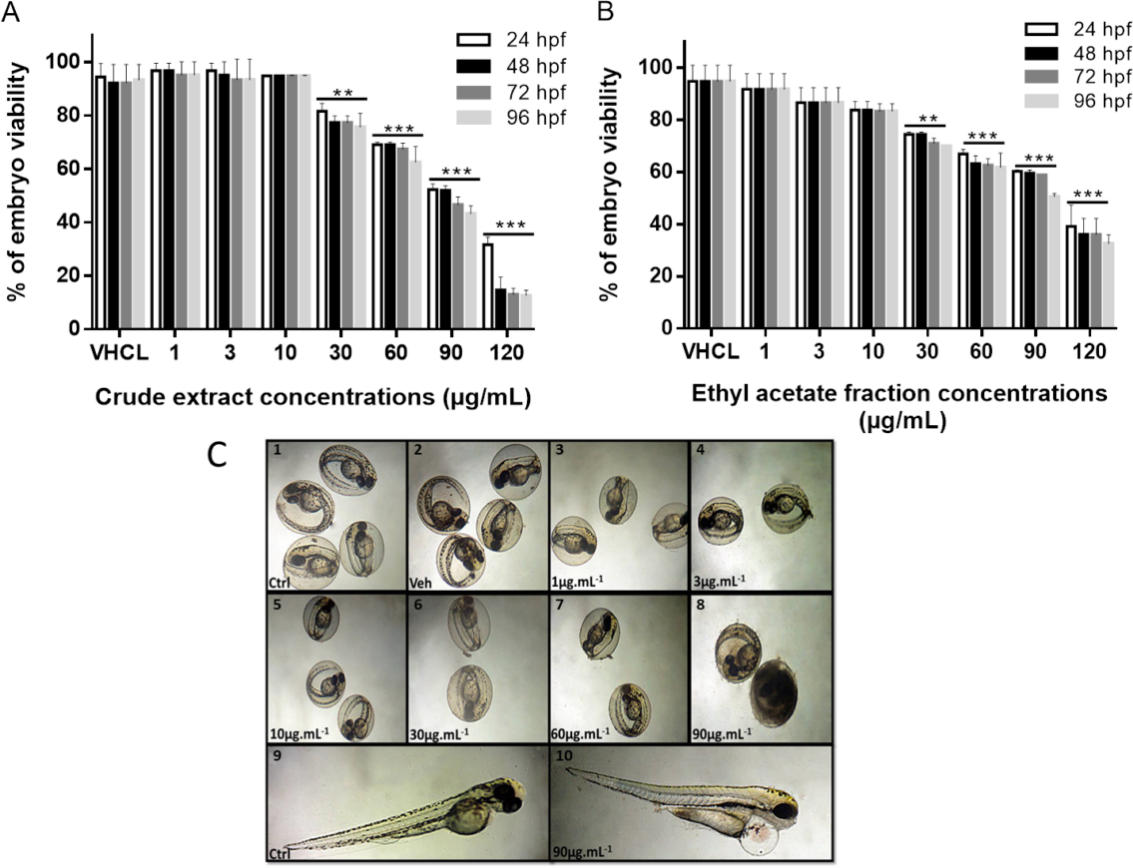
Viability of zebrafish embryos after exposure to the CE and EtOAc fraction at 24 h, 48 h, 72 h and 96 h post treatment. Effect of crude extract (A) and Ethyl acetate fraction (B) on zebrafish embryos, viability rate after treatment. Mean values (% of control) are indicated. This experiment was performed in duplicates and repeated three times. ^∗∗^*P* < 0.01, ^∗∗∗^*P* ≤ 0.001. (C) Morphological alteration of zebrafish embryos after exposure to sumac CE. (1–10): embryos at 72 hpf, 1: Untreated, 2: DMSO, 3 : 1 μg/mL, 4: 3 μg/mL, 5: 10 μ g/mL, 6: 30μg/mL, 7: 60 μg/mL, 8: 90 μg/mL, Coagulation, 9: Untreated at 96 hpf, 10 : CE 90 μ g/mL, the arrow indicates pericardial edema. Scale bars 500 μ m (1–8), 250 μ m (9–10).

We have next tested the cytotoxic effect of sumac extracts *in vivo* on zebrafish embryos. Lethality of zebrafish embryos treated with sumac CE and the EtOAc fraction was defined when embryos showed coagulation and no visual heartbeat. The viability of embryos after 24, 48, 72 and 96 h post-fertilization (hpf) of exposure to different concentrations of the extracts are shown in Fig. 7. At 24 hpf, normal morphological development was observed with the presence of tail, head, eye and embryonic movement. Approximately, 30% of eggs coagulated after treatment with CE extract (60 µg/mL) and EtOAc fraction (60 µg/mL). Embryos coagulation was concentration-dependent showing a high mortality at a concentration of 120 µg/mL for CE extract and EtOAc fraction (Figs. 7A–7C). Coagulation usually happens naturally in 4–5% of fertilized eggs.

**Figure 8:**
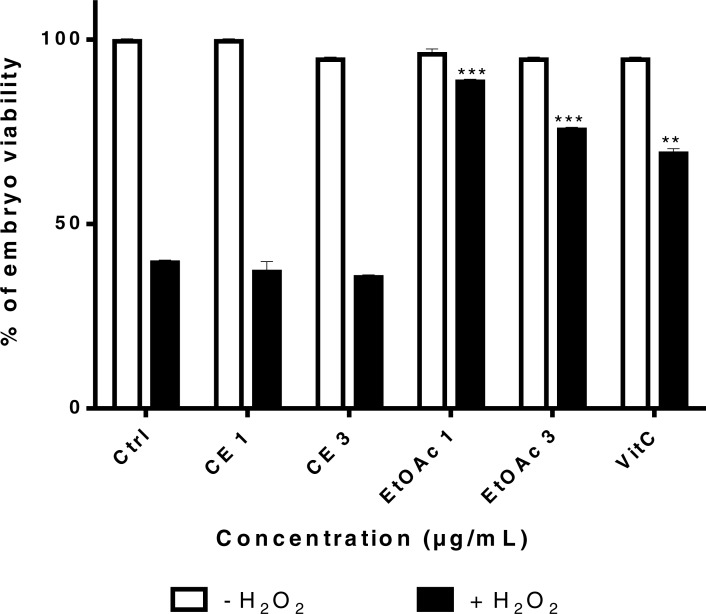
Viability rates of zebrafish embryos 24 h after treatment with different concentrations of sumac’s CE (1 and 3 μg/mL) extract, EtOAc fraction (1 and 3 μg/mL) and VitC followed by exposure to H_2_O_2_ for 2 h. The significance (^∗∗^*p* ≤ 0.01 & ^∗∗∗^*p* ≤ 0.001) of embryos viability in treated cells/ +H_2_O_2_ with respect to the untreated cells/ −H_2_O_2_.

No morphological abnormalities were seen at 48 hpf. Likewise, somite showed similar formation and number when comparing control to treated embryos with EtOAc fraction and CE. However, heart edema was observed at 96 hpf in embryos treated with EtOAc fraction and CE at 90 and 120 µg/mL. The latter observation may be due to an increase in the heart rate of the embryos (Figs. 7C–10). The treatment with CE and EtOAc fraction at 60 µg/mL or higher concentrations affected significantly the hatchability of the eggs (Supplemental Information 2).

In order to determine whether sumac CE and EtOAc fraction have a protective effect on H_2_O_2_-treated embryos, survival assays were carried out. An H_2_O_2_ killing curve was generated after treatment of embryos with 2.10^−3^ mol/L, 2.10 ^−2^ mol/L and 2.10^−1^ mol/L of H_2_O_2_ for 60 min at 24 hpf. The H_2_O_2_ concentration of 2.10 ^−2^ mol/L was chosen for further studies; it corresponds to ∼50% survival of embryos (Supplemental Information 2).

Embryos were pretreated at 4–6 hpf with different concentrations of CE, EtOAc fraction (1 and 3 µg/mL) and VitC (100 µM) for 24 h and then exposed to 2.10^−2^ mol/L H_2_O_2_ or the E3 medium (control) at 24 hpf for 2 h. Treatment with the EtOAc fraction treatment at low concentrations (1 and 3 µg/mL) increased the survival rate of H_2_O_2_-treated embryos relatively to untreated embryos (Fig. 8). Sumac CE failed to increase the viability of H_2_O_2_-treated embryos

## Discussion

Natural compounds from plants are an extremely important source of medicinal agents. A recent qualitative study of the phytochemical components from sumac fruit extract showed that this spice is an abundant source of bioactive molecules. It contains phenolic acids, flavonoids, iso-flavonoids, tannins, anthocyanins, etc. (Abu-Reidah et al., 2015). Here we have studied the antioxidant effect of *Rhus coriaria* extracts. First, we used DPPH scavenging and β-carotene bleaching assays to show that CE extract and the EtOAc fraction exhibit the greatest antioxidant activity compared to the positive controls (catechin and ascorbic acid). These results were consistent with others study in the literature. Indeed, Jamous et al. (2015) showed the antioxidant potential of *Rhus coriaria* methanolic extract using the DPPH scavenging test. A study of the antioxidant activity of water sumac extract compared to other plants extracts and showed that sumac possessed the highest antioxidant potential (Aliakbarlu, Mohammadi & Khalili, 2014). Moreover, the aqueous and alcoholic extracts of *R. coriaria* were good scavengers for ROS and are a potential source of natural antioxidants for use in pharmaceutical or food industry (Al-Muwaly, Al-Flayeh & Ali, 2013). Many other studies indicated that the antioxidant activity of sumac extracts might result from polyphenolic constituents, especially gallic acid and its derivatives (Chakraborty et al., 2009; Ferk et al., 2007; Gabr, El-Metwally & Al-Ghadir, 2014). The antioxidative activity displayed by sumac extract and fractions are due to phenolic compounds and as shown by many studies, there was substantial relationship observed between total phenols and antioxidant activity (Kosar et al., 2007). All the phenols in CE extract and EtOAc fraction might act additively and even synergistically that subsequently increasing the antioxidant activity. Our results showed a protective antioxidant effect of gallic acid at low concentration; however, it is cytotoxic at high concentrations. It was mentioned that Gallic acid provokes DNA damage and suppresses DNA repair gene expression in human prostate cancer PC-3 cells (Liu et al., 2013). This cytotoxic activity of gallic acid might explain why the sumac cytotoxicity increases at high concentrations. Consequently, the crude extract or the EtOAc fraction can be utilized as an effective natural antioxidant source.

Here we have identified a new function for *Rhus coriaria*. The ethyl acetate fraction of *R. coriaria* has the ability to protect muscle satellite cells from induced oxidative stress. Furthermore, we have shown for the first time that sumac extracts had the same protective effect *in vivo* on zebrafish embryos. Indeed, EtOAc fraction improved survival and protected myoblasts and zebrafish embryos from oxidative stress. This fraction significantly reduced cell cycle arrest in myoblasts subjected to oxidative stress. It also protected human myoblasts from H_2_O_2_-induced oxidative stress by increasing the expression of the SOD2 and catalase. Moreover, it might delay muscle differentiation by decreasing myogenin expression.

Riederer et al. (2012) reported that, 3 days after transplantation, myoblasts undergo differentiation, hence limiting their ability to proliferate. Our results indicate that sumac extract decreases myogenic markers’ expression, which may result in delayed differentiation. This supports a function for sumac extract to enhance cell survival and proliferation concomitant with a delay in differentiation during this critical time window of 3 days post-implantation, allowing more efficient skeletal muscle regeneration.

The role of oxidative stress in muscle pathology (Canton, Menazza & Lisa, 2014; Terrill et al., 2013) was implicated early by the observation that muscles from Duchenne Muscular Dystrophy patients contain a higher level of thiobarbituric acid reactive products, which was indicative of lipid peroxidation brought about by oxidative stress (Kar & Pearson, 1979). Moreover, muscle cells from FSHD patients show increased susceptibility to oxidative stress, augmented lipofuscin inclusions, elevated expression of antioxidant enzymes, dysfunctional mitochondria (Turki et al., 2012) and high levels of DNA damage (Dmitriev et al., 2016).

Myoblast transplantation represents a viable approach for the treatment of myopathies associated with fiber necrosis and muscle weakness (Gussoni, Blau & Kunkel, 1997). Transplanted myoblasts can fuse with endogenous muscle fibers to form myotubes (Partridge et al., 1989). However, cell-based therapy for skeletal muscles degenerative diseases is still disappointing. There is evidence that oxidative stress, which is presumably derived from damage resulting from intramuscular implantation, might cause rapid cell death and hence poor outcome. The enhancement of cell survival by protecting from oxidative stress highlights the need to find new and potent natural antioxidants to improve cell- based therapy for muscle diseases and to delay muscle aging.

Mature skeletal muscle cells, as well as myogenic stem and progenitor cells, elaborate sophisticated enzymatic antioxidant systems; this renders them extremely flexible in response to changes in redox potential (Beckendorf & Linke, 2015; Powers, Talbert & Adhihetty, 2011). The primary antioxidant enzymes in muscle cells include superoxide dismutase, glutathione peroxidase, and catalase. Dystrophic muscles exhibit enhanced catalase, SOD, and glutathione reductase activity, which is reflective of oxidative stress (Candan & Sökmen, 2004). A previous study showed that GPx3 plays a major role in human myoblast viability and mediates the anti-cytotoxic effect of RA (El Haddad et al., 2012). In our study, the levels of SOD2 and catalase were increased in cells treated with low concentrations of the EtOAc fraction, whereas this treatment induced a significant decrease in the expression of GPx3 mRNA. This result shows that SOD and catalase are implicated in the protective antioxidant effect of the EtOAc fraction.

This study is also the first complete assessment of the toxicity and antioxidant activity of sumac fruit extracts on zebrafish embryos. Zebrafish are widely used for *in vitro* assays in drug/pharmaceutical research (Alestrom, Holter & Nourizadeh-Lillabadi, 2006). The CE and the EtOAc fraction were screened for their effects on the development of zebrafish embryos. None of the extracts induced abnormal development of embryos at low concentrations. However, high concentrations were associated with developmental abnormalities in a dose-dependent manner. The embryos treated with high doses of sumac extracts showed cardiac edema with the enlarged cardiac chamber (cardiac hypertrophy). Furthermore, the embryo pretreatment with a low concentration of the EtOAc fraction protected zebrafish from H_2_O_2_-induced oxidative stress.

## Conclusion

Normal muscle cells and myoblast are both sensitive to oxidative stress. Hence, a comprehensive therapeutic approach to muscle atrophy should take into account the relative contribution of oxidative stress. Natural antioxidant treatment represents a promising strategy in the treatment of muscular pathologies by preventing oxidative injury and potentially delay disease progression.

Here we have found that crude and ethyl acetate fraction inhibited or slow down the progress of skeletal muscle atrophy by decreasing oxidative stress, thus playing a major role in the modulation of cells aging process. In myoblasts, these extracts can increase the viability of implanted skeletal muscle precursor. The current results are also encouraging for screening the effect of other medicinal plants on zebrafish embryos for drug discovery, biotechnological and medical applications. Therefore, further studies are required to gain more insights into the protective mechanisms of sumac extracts.

##  Supplemental Information

10.7717/peerj.4144/supp-1 Data S1 Raw data

10.7717/peerj.4144/supp-2 Supplemental Information 1 Cell cycle distribution of LHCN-M2 after treatment with sumac crude extract and ethyl acetate fraction Histogram showing the percentages of cells at various phases of cell cycle. Values are means of three independent experiments.

10.7717/peerj.4144/supp-3 Supplemental Information 2 Hatching of zebrafish embryos treated with sumac crude extract and ethyl acetate fraction at 96 hpf Hatching rates of the embryos were completed after 96 hpf of crude extract (A) and ethyl acetate fraction (B) exposure. This experiment was performed 3 times. The significance (^∗^*p* ≤ 0.05, ^∗∗^*p* ≤ 0.01 & ^∗∗∗^*p* < 0.001) of embryos hatching in exposed embryos with respect to untreated embryos (CTRL).

10.7717/peerj.4144/supp-4 Supplemental Information 3 Viability of zebrafish embryos after exposure to H_2_O_2_ Effect of H_2_O_2_ on zebrafish embryos viability after treatment with the following concentrations 10^−3^, 5. 10^−3^, 7.5 10^−3^, 10^−2^, 2.10^−2^, 2.5 10^−2^, 2.5 10^−2^, 3. 10^−2^, 5.10^−2^, 7.5 10^−2^, 10^−1^. Mean values with S.D. are indicated. This experiment was performed in duplicates and repeated 3 times.

## Abbreviations

Aq: Aqueous
CE: Crude extract
DPPH: free radical scavenging activity
DHE: Dihydroethidium
EtOAc: Ethyl acetate
Hex: Hexane
H_2_O_2_: Hydrogen peroxide
hpf: Hour post fertilization
IC_50_: Inhibition concentration
MD: Muscular disptrophies
ROS: Reactive oxygene species
SA: scavenging activity
VitC: Vitamin C

## Additional Information and Declarations

### Field Study Permissions

The following information was supplied relating to field study approvals (i.e., approving body and any reference numbers): As we already mentioned in the Material and Methods section, the fresh fruit from *Rhus coriaria L.* plant was collected from South Lebanon. No field permit was required because the fresh fruits were collected from a private field (that the corresponding author owns). Fresh fruit was only collected from the field and all experimental procedures were done in the laboratory.

### Data Availability

The following information was supplied regarding data availability: The raw data has been provided as a Supplemental File.
