# Supplementary figures and images for: Protective effect of Rhus coriaria fruit extracts against hydrogen peroxide-induced oxidative stress in muscle progenitors and zebrafish embryos
Source: PeerJ. 2017 Dec 12;5:e4144. doi: 10.7717/peerj.4144 (PMC5731335; doi:10.7717/peerj.4144)

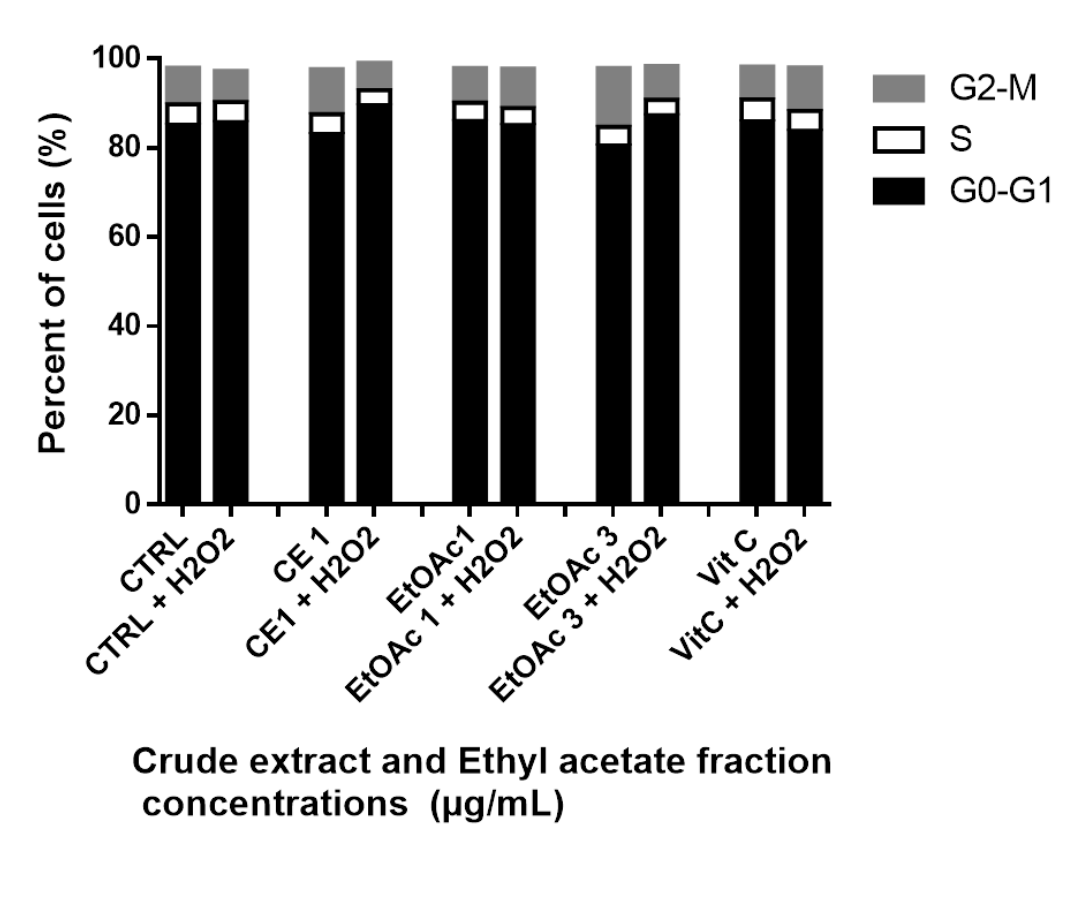

Supplement: Supplemental Information 1 — Histogram showing the percentages of cells at various phases of cell cycle. Values are means of three independent experiments. [file peerj-05-4144-s002.png]

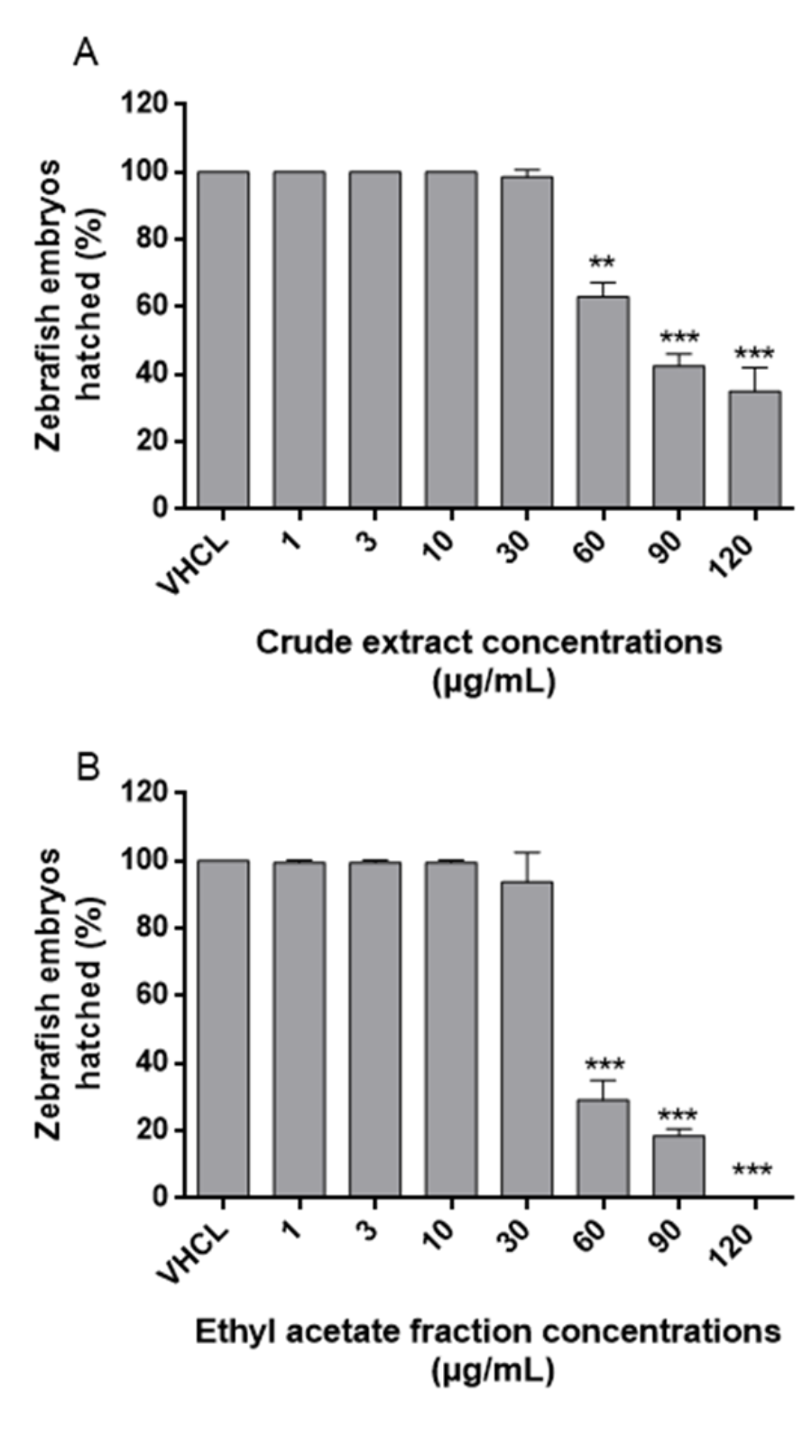

Supplement: Supplemental Information 2 — Hatching rates of the embryos were completed after 96 hpf of crude extract (A) and ethyl acetate fraction (B) exposure. This experiment was performed 3 times. The significance (∗p ≤ 0.05, ∗∗p ≤ 0.01 & ∗∗∗p < 0.001) of embryos hatching in exposed embryos with respect to untreated embryos (CTRL). [file peerj-05-4144-s003.png]

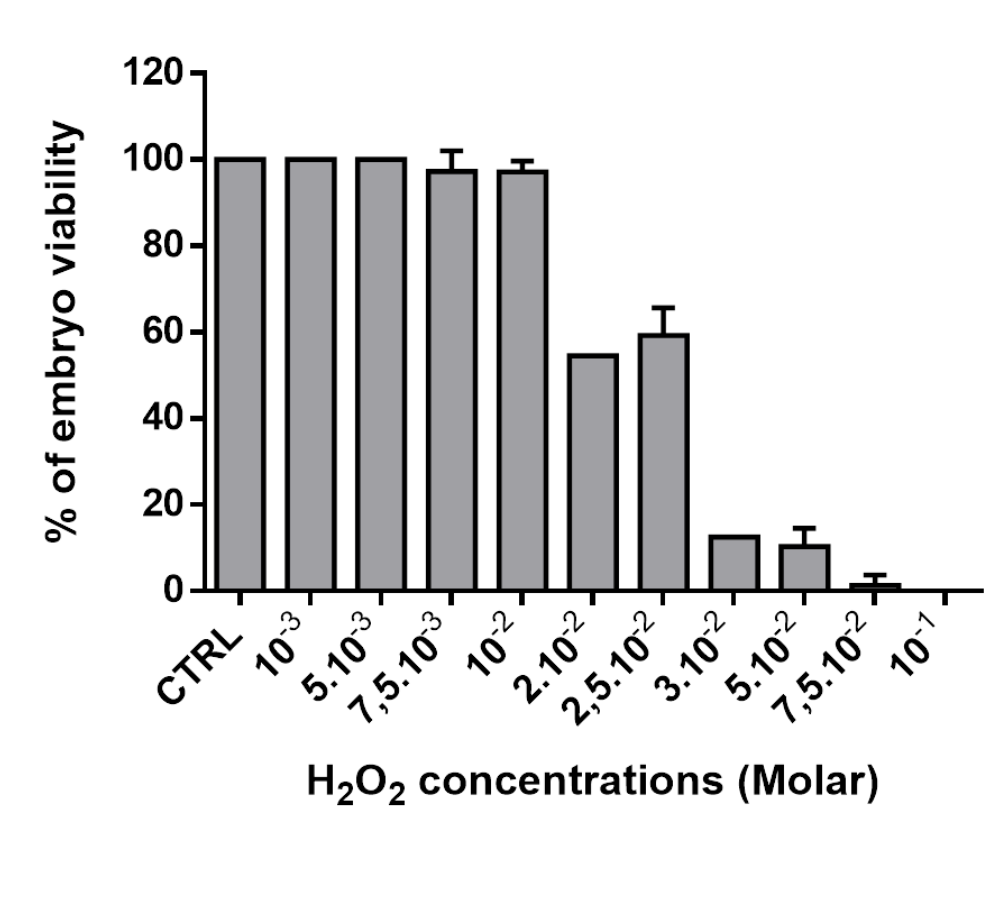

Supplement: Supplemental Information 3 — Effect of H2O2 on zebrafish embryos viability after treatment with the following concentrations 10−3, 5. 10−3, 7.5 10−3, 10−2, 2.10−2, 2.5 10−2, 2.5 10−2, 3. 10−2, 5.10−2, 7.5 10−2, 10−1. Mean values with S.D. are indicated. This experiment was performed in duplicates and repeated 3 times. [file peerj-05-4144-s004.png]
